# A framework for inferring and analyzing pharmacotherapy treatment patterns

**DOI:** 10.1186/s12911-024-02469-4

**Published:** 2024-03-08

**Authors:** Everett Rush, Ozgur Ozmen, Minsu Kim, Erin Rush Ortegon, Makoto Jones, Byung H. Park, Steven Pizer, Jodie Trafton, Lisa A. Brenner, Merry Ward, Jonathan R. Nebeker

**Affiliations:** 1 Oak Ridge National Laboratory, Oak Ridge, TN USA; 2 University of New Mexico School of Medicine, Albuquerque, NM USA; 3 US Department of Veterans Affairs, Washington DC, USA; 4 School of Medicine, University of Utah, Salt Lake City, UT, USA; 5 VA Boston Healthcare System, Boston, MA USA; 6 Palo Alto VA Healthcare System, Palo Alto, CA USA; 7 VA Rocky Mountain Mental Illness Research, Education and Clinical Center, Aurora, CO USA

**Keywords:** Process mining, Clinical pathways, Major depressive disorder

## Abstract

**Background:**

To discover pharmacotherapy prescription patterns and their statistical associations with outcomes through a clinical pathway inference framework applied to real-world data.

**Methods:**

We apply machine learning steps in our framework using a 2006 to 2020 cohort of veterans with major depressive disorder (MDD). Outpatient antidepressant pharmacy fills, dispensed inpatient antidepressant medications, emergency department visits, self-harm, and all-cause mortality data were extracted from the Department of Veterans Affairs Corporate Data Warehouse.

**Results:**

Our MDD cohort consisted of 252,179 individuals. During the study period there were 98,417 emergency department visits, 1,016 cases of self-harm, and 1,507 deaths from all causes. The top ten prescription patterns accounted for 69.3% of the data for individuals starting antidepressants at the fluoxetine equivalent of 20-39 mg. Additionally, we found associations between outcomes and dosage change.

**Conclusions:**

For 252,179 Veterans who served in Iraq and Afghanistan with subsequent MDD noted in their electronic medical records, we documented and described the major pharmacotherapy prescription patterns implemented by Veterans Health Administration providers. Ten patterns accounted for almost 70% of the data. Associations between antidepressant usage and outcomes in observational data may be confounded. The low numbers of adverse events, especially those associated with all-cause mortality, make our calculations imprecise. Furthermore, our outcomes are also indications for both disease and treatment. Despite these limitations, we demonstrate the usefulness of our framework in providing operational insight into clinical practice, and our results underscore the need for increased monitoring during critical points of treatment.

**Supplementary Information:**

The online version contains supplementary material available at 10.1186/s12911-024-02469-4.

## Background

Depression is the single largest contributor to disability worldwide [1]. Major depressive disorder (MDD) is the most common form of depression [2]. Between 2010 and 2019, the number of adults with at least one major depressive episode in the prior 12 months increased 25.2%, from 15.5 million to 19.4 million [3, 4]. While the cost of prescription drugs has decreased over time, the direct costs incurred by those with MDD rose 2.8% between 2010 and 2018 with medical services costs growing 18.1% and suicide-related costs growing 22.8% [3].

Veterans from Operation Enduring Freedom (OEF) and Operation Iraqi Freedom (OIF) have higher percentages of major depressive episodes than non-veterans of the same age [5]. In response, the Department of Veterans Affairs (VA) has decreased suicides, improved drug monitoring programs, and increased population coverage by expanding mental health resources and by focusing existing resources on clinical practices that make the most difference [6–10]. Existing reports and dashboards at VA cannot support policymakers with enough detailed insight into the full array of patient-level clinical treatment pathways to guide corrective action and resource provisioning efforts [11–16].

With the increasing availability of electronic health records (EHRs) and advances in data analytics, opportunities to build data-driven approaches to infer evidence-based practice patterns have grown [17]. EHRs are patient-centric and real-time records, including treatment history of patients, such as laboratory services, procedures, inpatient/outpatient medications, radiology, nuclear medicine services, and consultations [18], which can be used to obtain clinical insights and their evidences. The effort to understand procedural patterns and optimize their performance is not unique to healthcare. In industry, process mining, a relatively young research discipline but already with notable achievements, automatically identifies procedures used in an organization (e.g., manufacturing or financial) and compares them against proposed procedures, which enables optimization with respect to given outcomes through iterative adjustments. When applied to healthcare, it has proven to find practice patterns as a collection of executions of healthcare processes, where each process is a sequence of clinical activities conducted to diagnose, treat, and evaluate conditions [13, 19–21]. In fact, acquiring process models close to the real practice patterns and evaluating their efficacy using evidence in EHR helps identify better treatment options, necessary collaboration between healthcare system and patients, and eventually redesign of the clinical pathway.

Despite its great potential, applying process mining to infer clinical pathways faces several issues. First, process mining assumes availability of structured data that contains records and timestamps of events (or activities) so called an event log. This implies there should exist a set of well defined medical events, and all EHRs of the patients in the cohort are mapped to the events and arranged in the chronological order. However, to create such mappings, multiple professionals with a wide range of expertise within healthcare domain should together extract appropriate data elements from EHR and associate them with well-defined clinical events. This is, however, not an easy task. Domain experts typically have limited views on the clinical pathways beyond their specialty. Moreover, EHR data are known to have missing, incorrect, imprecise, and irrelevant elements to apply processing mining [22]. There exist some works that try to automate this process. For example, a framework that generates an event log and feeds it to pMineR [23] to generate a process model was introduced by [24]

This study presents a data-driven framework for providing explainable insights and the ability to find critical decision points in the treatment of MDD with antidepressants. We present results of the framework using 15 years of pharmacy and administrative data on OEF/OIF veterans from VA’s Corporate Data Warehouse (CDW). Our approach uses state-of-the-art process mining techniques to describe prescription patterns and sequential rule mining to evaluate complex treatment patterns. Additionally, we propose a novel preprocessing technique that abstracts pharmacy data into both a trace suitable for process mining and a sequence database suitable for sequential rule mining.

## Methods

**Fig. 1:**
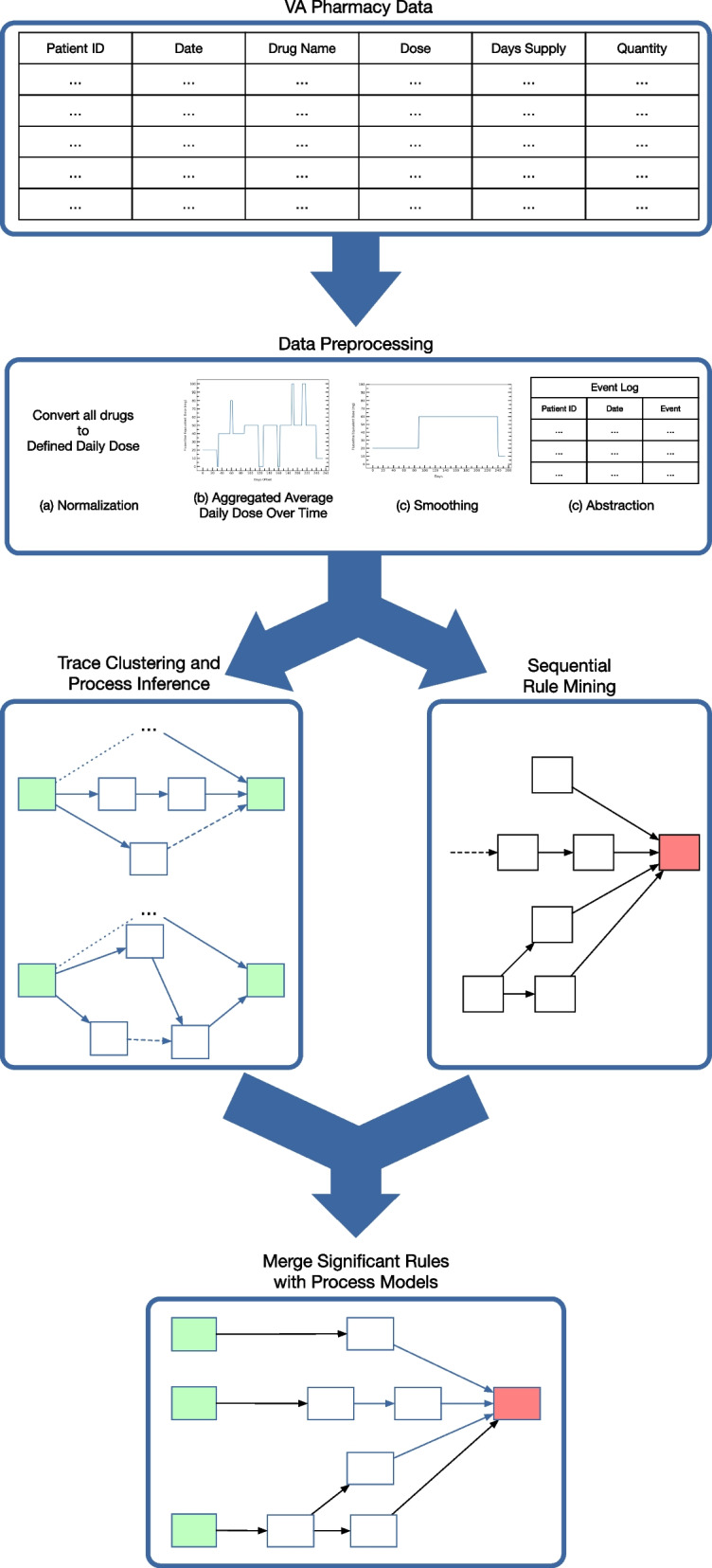
This is an overview of Inference and Analysis Framework

Our four-step framework takes pharmacy data and adverse events as input. 1) The preprocessing step normalizes all drug dosages to their fluoxetine equivalents, applies median smoothing to the prescribed daily dose, and abstracts the data into clinical actions. 2) We collect all the clinical actions for each depressive episode into a trace and apply agglomerative clustering to subdivide individuals based on their treatment pathway. Using these clusters, we formulate process inference as an optimization problem. 3) Additionally, we store the clinical actions for each depressive episode as a sequence database suitable for discovering statistically interesting rules correlated with adverse events. 4)We merge the discovered process models and association rules into a single process model. Figure 1 provides an overview of our framework.

### Data sources and preprocessing

The CDW contains inpatient and outpatient pharmacy information for 12 million individuals aggregated from over 130 VA facilities across the United States. In this study, we used CDW data between January 1, 2006 and January 1, 2020 [25]. We created a cohort of veterans that were diagnosed with MDD and served during OEF or OIF. This cohort contains younger veterans that do not require the special considerations of geriatric populations. We considered a positive diagnosis of MDD as either one inpatient diagnosis or two outpatient diagnoses of an MDD ICD9 (296.20 – 296.26) or ICD10 code (F32.0 – F33.9). We used VA stop code 130 to determine emergency department visits, the self-directed violence classification system for self-harm [see Additional file 1], and the date of death from the CDW for all-cause mortality [26, 27].

We extracted pharmacy data for the most common antidepressants prescribed at the VA in both inpatient and outpatient settings, i.e., sertraline, citalopram, fluoxetine, escitalopram, paroxetine, venlafaxine, duloxetine, trazodone, nefazone, and bupropion. These drugs comprise a majority of the antidepressant prescription fills for the MDD cohort. After extracting the data, the pharmacy records were cleaned to ensure that dosage, days supply, and quantity were in acceptable ranges. We computed histograms for each drug and variable removing any value that was an outlier for that drug. An outlier is defined as a value that occurs in <0.1% of fills for a given drug. After cleaning, the SMEs validated that the data ranges were appropriate for each drug.

We normalize all drug doses into their fluoxetine equivalent so that we can compare prescription patterns across drugs. While there are several conversion algorithms, we chose a combination of two methods: those of Hayasaka et al. and the Defined Daily Dose (DDD) from the World Health Organization. Hayasaka et al. use double-blind flexible dose trials to compute the optimal mean dose for each drug [28, 29]. On the other hand, the DDD is based on the drug’s product information sheet [30, 31]. Table 1 presents the drug equivalents used in this study. We adopt the dose equivalencies from Hayasaka et al. where one exists and use the defined daily dose otherwise.

**Table 1:** For each drug in the study, this table provides equivalent milligrams between drugs

Drug	Defined daily dose	Hayasaka et al. (2015)	Current study
Sertraline	50.0	49.3	49.3
Citalopram	20.0	-	20.0
Fluoxetine	20.0	20.0	20.0
Escitalopram	10.0	9.0	9.0
Paroxetine	20.0	17.0	17.0
Venlafaxine	100.0	74.7	74.7
Duloxetine	60.0	-	60.0
Trazodone	300.0	200.7	200.7
Nefazodone	400.0	267.6	267.6
Bupropion	300.0	174.25	174.25

The next preprocessing step approximates the milligrams of antidepressants that are consumed each day. For a given prescription, we use the fluoxetine equivalent dosage, days supply and quantity to determine the prescribed daily dose. Days with overlapping prescriptions are added together when a fill is released before the days supply of another prescription is over. Similar techniques are used in Coupland et al. [32, 33]. We call the resulting metric the normalized prescribed daily dose.

The normalized prescribed daily dose (PDD) can vary widely if a prescription is filled early or late. Gaps between prescriptions are treated as days with 0 mg PDD, and fills that are early are added on overlapping days. We smooth the PDD curve by binning and using the median filter with a sliding window. Binning is a common approach used in previous studies [32–34]. We binned average daily dose into five categories: <20mg, 20-39mg, 40-59mg, 60-80mg, and >80mg. The size of the sliding window is calculated on a per person basis. We calculated the average days supply of all prescriptions filled by an individual. Note that we only consider the fills of drugs listed in Table 1 in this calculation. After obtaining an individual’s PDD and sliding window size, we apply the median function to smooth each data point on the curve.

Finally, we transformed each smoothed curve into a sequence of actions suitable for pathway inference. The action space mirrors actions from the Texas Medication Algorithms Project [35]. Our action space N is start drug, increase dose, decrease dose, and continue at the current dosage. The start drug event occurs at the beginning of an episode when a drug is first started or the first prescription fill after 180+ consecutive days of no fills. This technique is also used to preprocess inputs to the REACH VET medication adherence algorithm [36]. The increase and decrease dose action corresponds to change from one bin to another with higher or lower dosage, respectively. The continue at current dose action specifies a duration of time over which the dosage stays constant.

### Trace clustering

Antidepressant prescription patterns will vary based on the guidelines or algorithms being followed by the prescriber. There are a variety of MDD guidelines and algorithms available that can differ in significant ways [37]. For example, there is disagreement on the second and third-line medications for MDD [2, 38, 39]. Some guidelines suggest changing class while others suggest the use of tricyclic antidepressants. This variety in recommendations from guidelines leads to a multiplicity of guideline compliant prescription patterns.

Trace clustering is an effective technique to divide large complex logs into coherent groups that share similar characteristics [40]. Many approaches build process models and cluster the process models or cluster individual traces directly. Current approaches work directly with traces and represent each trace as a vector that can be easily clustered [41–44]. We prefer to represent each trace t as a matrix $T_{nxn}$ where *n* is the cardinality of action space *N* and $T_{ij}=1$ if action $n_j$ directly follows action $n_i$ in t. We use the Frobenius norm to calculate the distance $\left\| A-B\right\| _{F}$ between each pair of matrices, *A* and *B*. Then we use agglomerative hierarchical clustering to cluster similar traces [40].

### Process inference

The collection of all actions for a single medication episode is a trace. The collection of all traces across the entire cohort is called an event log. Given an event log, we wanted to find the process model *PsM* with bounded complexity that performs best under a given metric. A *PsM* is defined by a set of nodes and edges such that $PsM=(N,E)$ where |*E*| is fixed. For $n \in N$, *n* is an action from the event log. For a given edge $(n_i,n_j) \in E$, the edge denotes that $n_i$ occurs directly before $n_j$ in the *PsM*.

The replayability game scores each *PsM* based on a given replayability function *R* over a given log *L*. The replayability score of an individual trace $\sigma \in L$ using *PsM* is $R(PsM,\sigma )$. Prodel et al. introduces eight replayability functions with varying properties [45]. The *R* function gives the percentage of events replayed with a penalty for skipping events. The *R* function is defined as

1 $$\begin{aligned} R(PsM, \sigma ) = \left( \frac{L(Psm,\sigma )}{n} - \alpha \delta (PsM,\sigma ) \right) ^{+} \end{aligned}$$

where $L(PsM,\sigma )$ denotes the cardinality of the longest subsequence of trace $\sigma$ that can be replayed using *Psm*, $\alpha$ is a constant, and $\delta$ is a binary indicator variable activated when an event is skipped. The *R* score is then averaged across all traces in the event log.

We used a heuristic algorithm to search for a *PsM* that scored highest with respect to the *R* function. We utilized a Python implementation of Tabu Search where the local moves are based on edge frequency. The tabu list is first-in first-out with a fixed size of 20. This methodology has been shown to navigate the search space well and outperform existing methodologies when modelling the complex processes often seen in medical data [45].

### Sequential rule mining

Sequential Rule Mining is a data mining technique used to extract sequential patterns from a sequence database. A sequential rule has the form $X \Rightarrow Y$. The rule $X \Rightarrow Y$ is read if a sequence of events *X* occurs then another sequence of events *Y* is likely to occur. Two definitions for sequential rule mining exist, and we adopt the same definition of sequential rules as Fournier-Viger et al. [46–48]. Formally, there is a set of sequences $S = \{ s_1, s_2, ... ,s_n \}$ and a set of items $I = \{ i_1, i_2, ... , i_m \}$. A sequence $s_i$ is an ordered list of itemsets $s_i = \{ I_1, I_2, ... , I_t \}$. For two unordered itemsets $X, Y \subseteq I$, a sequential rule states that if the items of *X* occur in a sequence, then the items in *Y* will occur afterward in the same sequence.

We use different statistical measures to assess the interestingness of the sequential association rules. For each sequential association rule $X \Rightarrow Y$ we capture support, confidence, and odds ratio. Support is defined as *P*(*X*, *Y*). Confidence is defined as *P*(*Y*|*X*). Since adverse events happen with very small frequency, support and confidence do not describe the sequential rules well. To remedy this problem, we also report the odds ratio as

2 $$\begin{aligned} OR = \frac{P(X,Y)P(\overline{X},\overline{Y})}{P(\overline{X},Y)P(X,\overline{Y})} \end{aligned}$$

previously defined in Tan et al. [49]. A high odds ratio metric denotes that a sequence containing *X* is more likely to have *Y* than sequences without *X*.

### Merge rules with clinical context

The sequential rules themselves do not provide enough context around mined associations. For example, an association that happens frequently after months of treatment is different than one found at the very beginning of treatment. This step uses a bow-tie analysis where we consider events within a specific time frame of a rule of interest to generate enough clinical context to understand the association rule [14].

First, we create a log with the traces associated with a sequential rule of interest. We truncate each trace to only consider events within a uniform amount of time of the adverse event. Using the time filtered log, we perform process inference. We visualize the clinical context using a custom pathway visualization tool on our Github page [50].

## Results

The OEF/OIF MDD cohort has 457,697 individuals with an average age of 41.7 years. We found antidepressants in VA pharmacy records for 252,179 out of the 457,697 individuals from the OEF/OIF MDD cohort (55%). We applied our data preprocessing methodology and created 288,344 antidepressant medication traces excluding any trace starting within 6 months of the data cutoff date of January 1, 2020. During treatment with antidepressants there are 98,417 cases of emergency department visits, 3,928 cases of self-harm, and 1,507 deaths from all causes. Table 2 provides a summary of the data.

**Table 2:** This is a summary table that includes the total number of traces, outcomes, and demographics

Outcomes		
	Total Traces	288,344
	ED Visits	98,417
	Self-Harm	1,016
	All-cause Mortality	1,507
Characteristic		
Race		
	White non-Hispanic	274,879
	Black non-Hispanic	84,233
	Hispanic	57,529
	Other	41,056
Gender		
	Female	388,006
	Male	69,691
Priority Group		
	1	233,682
	2	37,545
	3	35,064
	$\ge$4	151,869

### Trace clustering and process inference

After trace clustering and process inference, we had 3,607 different clusters each with their own process model. We achieved an average *R* score of 0.891 across all traces.

**Fig. 2:**
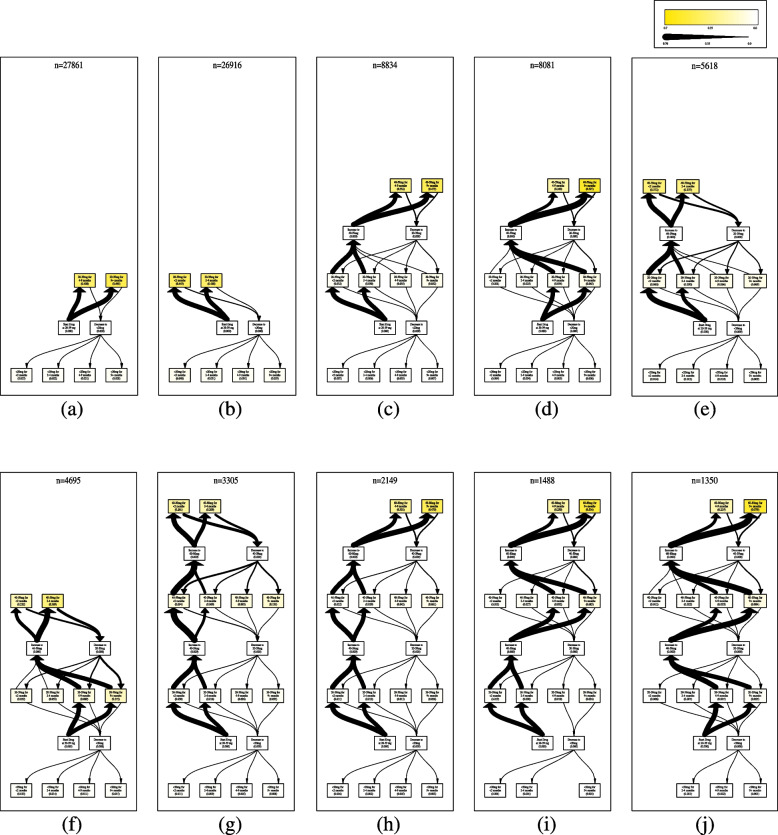
This figure shows the top 10 process models that start at 20-39mg. The value in each box represents the percentage of people whose treatment stopped with this activity. The thickness of arrows is a normalized edge weight with edge frequency divided by *n*

The process models from the 10 largest clusters in each bin completely replay 69.3% of all traces which we present in Fig. 2 (top 10 process models for traces that start at 20-39mg). These 10 process models completely replay 90,297 of the 130,207 traces. Figure 2 provides a visualization of each process model along with the number of traces completely replayed on the process model. The images are sorted in descending order based on the number of traces that can be completely replayed. For example, Fig. 2a shows the most common prescription pattern. Figure 2a shows that the normalized prescribed daily dose stays in the 20-39mg dose range for 4 or more months with some discontinuing at a lower dose or staying on the lower dose long-term.

### Sequential rule mining

We created sequences by dividing up the smoothed prescribed daily dose curve into two-week increments. We choose two week increments by looking at the distribution of the amount of time from start or dosage change to adverse event and two weeks was a good cut-off point. Additionally, this enables us to compare our results with existing literature that suggests adverse drug reactions occur within the first two weeks after dosage change [51]. Then we inserted the first adverse event into each sequence. We considered emergency department visits, documented self-harm, and all-cause mortality. After mining sequential association rules using window size of 1, 2, and 3, we kept rules with support greater than 10 and an odds ratio greater than 2.0.

Table 3 presents the top 10 sequential association rules for emergency department visits and self-harm. Length one rules were mined using a window size of 1, and length two rules were mined with window size of 2. There were no rules that met the support and odds ratio thresholds. Each section is sorted by increasing odds ratio. There were no significant length two rules found for self-harm and all-cause mortality. Note that these association metrics do not claim that there is a casual relationship between outcome and treatment.

**Table 3:** This provides sequential association rule metrics for associations between time duration, dosage, and outcomes

Outcome	Rule X$\Rightarrow$Y	Conf.	Supp.	Supp(X)	Crude OR
Emergency Dept. Visit					
	40-59mg 2-4 weeks	0.015	2,438	158,871	2.26
	<20mg 2-4 weeks	0.015	1,731	109,011	2.33
	80+mg 0-2 weeks	0.016	939	56,714	2.42
	20-39mg 2-4 weeks	0.017	3,188	191,807	2.46
	20-39mg 4-6 weeks	0.017	2,863	170,754	2.48
	<20mg 4-6 weeks	0.018	1,666	91,833	2.67
	60-80mg 0-2 weeks	0.019	1,796	95,595	2.77
	<20mg 0-2 weeks	0.020	2,262	114,732	2.92
	40-59mg 0-2 weeks	0.021	3,441	164,099	3.13
	20-39mg 0-2 weeks	0.021	4,144	198,259	3.14
	Length Two Associations				
	20-39mg 4-6 weeks and 60-80mg 0-2 weeks	0.021	36	1,742	2.11
	<20mg 2-4 weeks and 40-59mg 0-2 weeks	0.022	31	1,390	2.28
	20-39mg 2-4 weeks and 60-80mg 0-2 weeks	0.025	33	1,339	2.52
Self-Harm					
	<20mg 4-6 weeks	<0.0005	<20	91,833	2.28
	60-80mg 8-10 weeks	<0.0005	<20	74,958	2.79
	40-59mg 2-4 weeks	0.0002	31	158,871	2.97
	60-80mg 2-4 weeks	0.0002	20	91,618	3.06
	80+mg 0-2 weeks	<0.0005	<20	56,714	3.70
	80+mg 8-10 weeks	<0.0005	<20	45,015	3.72
	80+mg 2-4 weeks	<0.0005	<20	54,856	3.83
	40-59mg 0-2 weeks	0.0003	51	164,083	4.47
	80+mg 4-6 weeks	<0.0005	<20	51,995	4.86
	60-80mg 0-2 weeks	0.0004	37	95,595	5.52
All-cause Mortality					
	60-80mg 6-8 weeks	0.0003	20	79,250	2.37
	80+mg 2-4 weeks	<0.0005	<20	54,856	2.39
	60-80mg 4-6 weeks	0.0003	22	85,658	2.42
	80+mg 0-2 weeks	<0.0005	<20	56,714	2.48
	40-59mg 0-2 weeks	0.0003	44	164,083	2.55
	60-80mg 2-4 weeks	0.0003	26	91,618	2.68
	80+mg 10-12 weeks	<0.0005	<20	41,889	2.69
	80+mg 4-6 weeks	<0.0005	<20	51,995	2.71
	<20mg 0-2 weeks	0.0003	37	114,732	3.06
	60-80mg 0-2 weeks	0.0004	34	95,595	3.37

### Merge rules with clinical context

**Fig. 3:**
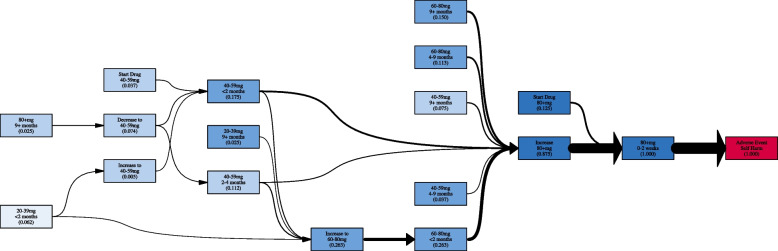
This image shows the clinical context for the sequential association rule (80+ mg 0-2 weeks$\Rightarrow$self-harm). The activities (boxes) are colored by dosage. The adverse event is highlighted in red. The thickness of each arrow represents the normalized edge frequency

Figure 3 shows the clinical context for the sequential association rule (80+ mg for 0-2 weeks$\Rightarrow$self-harm). This rule is interesting because it shows the recent history of individuals on a high dose of antidepressants (OR=3.70). The confidence metric for this rule shows 0.03% of individuals (n=17) that go above 80mg (n=56,716) have their first documented occurrence of self-harm within 0-2 weeks after increasing dosage.

## Discussion

We demonstrated that it is possible to describe a majority of antidepressant prescription patterns with a small number of process models. That is, 10 models accounted for almost 70% of prescription patterns. Furthermore, we derive new insights into critical points during MDD treatment. These insights can help inform drug monitoring efforts and suicidal behavior risk.

We found that 55.0% of the entire OEF/OIF MDD cohort took SSRIs or SNRIs at some point during their depressive episode. This is in line with national trends [52]. Most traces start initial dosing at either <20 mg (n=76,925) or 20-39 mg (n=130,207). The number of people at a suboptimal dose is much higher than the national average or 16% reported by Lou et al. Even after conducting a separate analysis using the FDA therapeutic dose definitions, the number of individuals starting at suboptimal doses remains high.

The replayability score of 0.891 shows that the discovered process models are able to replay more than 89% of the trace data. Our models were unable to incorporate low frequency events such as drastic dosage changes of +60mg or -60 mg. While we can include those transitions in the process models, the models are less interpretable and tend to look more like spaghetti. Therefore, we choose to restrict the number of edges in each process model in favor of more interpretable results with lower replayability scores.

Figure 2a-d account for a majority of the people that starts at 20-39mg. The most frequent prescription pattern is for people to stay in the 20-39mg range for more than 4 months. Figure 2b shows individuals discontinuing treatment in <2 months (n=11,924) or between 2-4 months (n=11,251). Figure 2c and d show almost equal numbers of people going up to the 40-59mg range for 4 or more months. Figure 2c spends <4 months at the 20-39mg range whereas Fig. 2d spends 4 or more months at 20-39mg. Figure 2c and h show a fast titration schedule going to higher doses of antidepressants with continued treatment for 4 or more months at the highest dosage. Figure 2d, i and j show a slow titration schedule that can happen over 4 or more months.

The treatment patterns for those individuals that start at higher doses are more varied than those that start at lower doses. The top 10 process models in the 20-39mg bin replay 69.3% of traces that start at 20-39mg. In contrast, the top 10 models in the 40-59mg range and 60-80mg range only replay 55.9% of traces and 50.5% of traces respectively.

The rules from Table 3 do not infer causation between treatment and outcome, but instead the rules capture the temporal order that events happen together. While Table 3 shows many rules occur in the first four weeks after starting treatment or a dosage change, we speculate that prescribers notice that treatment isn’t working, and they are trying to adjust the dosage. We also note that there is a possibility of capturing adverse drug reactions as well. Stübner et al. report that 93% of the suicidal adverse events from the European drug surveillance program occur within the first weeks of starting antidepressant medication or increasing dose [51].

Emergency department visits were the most frequent adverse events. The top four rules in this category all occur within the first two weeks of starting treatment or changing doses. There are only two rules identifying an association beyond 4 weeks. Interestingly, the length two associations all involve an increase in dose greater than 20mg. The self-harm outcomes has many of the same rules as emergency department visits. The top five rules all occur in the first two weeks after starting treatment or changing dose. Two rules occur after 4 weeks. The length two associations all occur in the first two weeks after a dosage change at high milligrams. Death from all causes is the lowest frequency outcome in the study with 1,507 deaths or approximately 0.6% of the cohort. The support for the top 10 rules sums to 15.8% of total number of deaths.

The sequential association rules must also be viewed in their clinical context. Figure 3 shows the clinical context for self-harm that happens within the first two weeks after going above 80mg. The rule is read as within 0-2 weeks after going above 80mg the first documented care of self-harm happens with OR=3.70. The average amount of time until the first documented self-harm event is 72.3 weeks. 11.75% of the traces that the rule applies to start at 80+ mg and within 2 weeks have a documented self-harm event. The other 76.5% were on a lower dose and increased to 80+ mg. For those that did not start at 80+mg, they either came from the 40-59mg range or the 60-80mg range. These results underscore the need for careful monitoring during these time periods.

This study has some limitations. First, observational data has sources of both biases and confounders. Indication bias is one large source of error. Indication bias arises because the outcomes of the study are indications of MDD and side-effects of treatment. Furthermore, we do not account for severity of depression or individuals with pre-existing conditions related to our outcomes. Next, the actual consumption of antidepressants is different than the prescribed daily dose. Consequently, we are unable to account for individuals that stock-pile medications and use them later.

Due to the limitations with our approach and data, we do not see this approach as a way to inform or influence clinical practice. We present this approach as a documentation and data exploration step that can provide a basis for further efforts into medication management. Additionally, there is potential to use the results from this framework in medication monitoring efforts.

## Conclusion

This study presented a data-driven framework for inferring pharmacotherapy treatment patterns and showcases a proof-of-concept study on pharmacotherapy prescription patterns used to treat MDD. Using pharmacy records for 252,179 individuals from an OEF/OIF cohort with MDD we documented and described the major pharmacotherapy prescription patterns implemented in the VA. We. We also added three outcomes to enable an association study between outcomes, drug dosage and treatment duration. We presented sequential association rules that link drug dosage and duration with outcomes. Then we presented a method for placing each rule in their clinical context for further investigation. Our results underscore the need for increased monitoring at certain points in pharmacotherapy treatment of MDD.

Our initial findings show that this is a promising approach for inferring and analyzing prescription patterns. We do not claim any causal relationship for our association rules. Future work is needed to perform a causal analysis between medication prescription patterns and outcomes.

### Supplementary Information

**Additional file 1.** Self-harm ICD9/ICD10 Definition File. This file contains the ICD9/ICD10 codes used to identify self-harm.

## Data Availability

The data underlying this manuscript cannot be shared publicly due to the privacy of individuals in the study. The data is stored inside a protected health data enclave at Oak Ridge National Laboratory.

## Abbreviations

CDW: Corporate Data Warehouse
DDD: Defined Daily Dose
MDD: Major Depressive Disorder
OEF: Operation Enduring Freedom
OIF: Operation Iraqi Freedom
PDD: prescribed daily dose
VA: Department of Veterans Affairs

## References

[1] James SL, Abate D, Abate KH, Abay SM, Abbafati C, Abbasi N (2018). Global, regional, and national incidence, prevalence, and years lived with disability for 354 diseases and injuries for 195 countries and territories, 1990–2017: a systematic analysis for the Global Burden of Disease Study 2017. Lancet..

[2] McQuaid JR, Buelt A, Capaldi V, Fuller M, Issa F, Lang AE, et al. The management of major depressive disorder: synopsis of the 2022 U.S. Department of Veterans Affairs and U.S. Department of Defense Clinical Practice Guideline. Ann Intern Med. 2022;175(10):1440–51. 10.7326/M22-1603. 10.7326/M22-1603 36122380

[3] Greenberg PE, Fournier AA, Sisitsky T, Simes M, Berman R, Koenigsberg SH (2021). The Economic Burden of Adults with Major Depressive Disorder in the United States (2010 and 2018). PharmacoEconomics..

[4] NIMH. 2019 NSDUH Annual National Report | CBHSQ Data. National Institute of Mental Health; 2019.

[5] National Academies of Sciences E, Division HaM, Services BoHC, Services CtEtDoVAMH. Clinical management of mental health conditions at the veterans health Adminstration. In: evaluation of the department of veterans affairs mental health services. National Academies Press (US); 2018. https://www.ncbi.nlm.nih.gov/books/NBK499504/. Accessed 9 Feb. 2022.

[6] Pickett T, Rothman D, Crawford EF, Brancu M, Fairbank JA, Kudler HS. Mental Health Among Military Personnel and Veterans. N C Med J. 2015;76(5):299–306. 10.18043/ncm.76.5.299. 10.18043/ncm.76.5.299 26946859

[7] Liu P, Combs A, Breland J, Trafton J, Harris AHS, Asch S, et al. Patient Race or Ethnicity, Health Care System Characteristics, and Community Factors Associated with Quality of Antidepressant Medication Management (<span style=“font-variant:small-caps;”>AMM</span> ). Health Serv Res. 2021;56(S2):66. 10.1111/1475-6773.13790.

[8] Lemke S, Boden MT, Kearney LK, Krahn DD, Neuman MJ, Schmidt EM (2017). Measurement-based management of mental health quality and access in VHA: SAIL mental health domain. Psychol Serv..

[9] Hepner KA. Quality of care for PTSD and depression in the Military Health System: phase I report. No. RR-978-OSD in Research report. Santa Monica: RAND Corporation; 2016. 10.7249/rhq-06-01-14 PMC5158278 28083442

[10] Hepner KA, Farris C, Farmer CM, Iyiewuare PO, Tanielian T, Wilks A, et al. Delivering clinical practice guideline-concordant care for PTSD and major depression in military treatment facilities. Rand Health Q. 2018;7(3). PMID: 29607247; PMCID: PMC5873520. 10.7249/rhq-07-03-03 PMC5873520 29607247

[11] Rotter T, Jong RBd, Lacko SE, Ronellenfitsch U, Kinsman L. Clinical pathways as a quality strategy. In: Improving healthcare quality in Europe: Characteristics, effectiveness and implementation of different strategies [Internet]. European Observatory on Health Systems and Policies; 2019. https://www.ncbi.nlm.nih.gov/books/NBK549262/. 31721544

[12] Lakshmanan GT, Rozsnyai S, Wang F. Investigating Clinical Care Pathways Correlated with Outcomes. In: Daniel F, Wang J, Weber B, editors. Business Process Management. vol. 8094. Berlin, Heidelberg: Springer Berlin Heidelberg; 2013. p. 323–338. http://link.springer.com/10.1007/978-3-642-40176-3_27.

[13] Rojas E, Munoz-Gama J, Sepúlveda M, Capurro D. Process mining in healthcare: A literature review. J Biomed Inform. 2016;61:224–36. 10.1016/j.jbi.2016.04.007. 10.1016/j.jbi.2016.04.007 27109932

[14] De Oliveira H, Prodel M, Lamarsalle L, Inada-Kim M, Ajayi K, Wilkins J, et al. “Bow-tie” optimal pathway discovery analysis of sepsis hospital admissions using the Hospital Episode Statistics database in England. JAMIA Open. 2020;3(3):439–48. 10.1093/jamiaopen/ooaa039. 10.1093/jamiaopen/ooaa039 PMC7660952 33215077

[15] Zhdanava M, Voelker J, Pilon D, Cornwall T, Morrison L, Vermette-Laforme M (2021). Cluster Analysis of Care Pathways in Adults with Major Depressive Disorder with Acute Suicidal Ideation or Behavior in the USA. PharmacoEconomics..

[16] Proudman D, Greenberg P, Nellesen D. The growing burden of major depressive disorders (MDD): implications for researchers and policy makers. PharmacoEconomics. 2021;39(6):619–25. 10.1007/s40273-021-01040-7. 10.1007/s40273-021-01040-7 PMC8134814 34013439

[17] Blumenthal D, Tavenner M. The, “meaningful use” regulation for electronic health records. N Engl J Med. 2010;363(6):501–4. 10.1056/NEJMp1006114 20647183

[18] Jha AK, DesRoches CM, Campbell EG, Donelan K, Rao SR, Ferris TG (2009). Use of electronic health records in US hospitals. N Engl J Med..

[19] van der Aalst WM (2012). Process mining: Overview and opportunities. ACM Trans Manag Inf Syst..

[20] van der Aalst WMP, van Dongen BF, Herbst J, Maruster L, Schimm G, Weijters AJMM (2003). Workflow mining: A survey of issues and approaches. Data Knowl Eng..

[21] van der Aalst WMP, Weijters AJMM. Process mining: a research agenda. Comput Ind. 2004;53(3):231–244. Process / Workflow Mining. 10.1016/j.compind.2003.10.001.

[22] Mans RS, Aalst Wvd, Vanwersch RJB. Process Mining in Healthcare: Evaluating and Exploiting Operational Healthcare Processes. Springer Publishing Company, Incorporated; 2015.

[23] Gatta R, Lenkowicz J, Vallati M, Rojas E, Damiani A, Sacchi L, et al. pMineR: An innovative R library for performing process mining in medicine. Artificial Intelligence in medicine. Cham: Springer International Publishing; 2017. p. 351–355.

[24] Gatta R, Vallati M, Lenkowicz J, Casà C, Cellini F, Damiani A, et al. A Framework for event log generation and knowledge representation for process mining in healthcare. 2018 IEEE 30th International Conference on Tools with Artificial Intelligence (ICTAI). 2018. p. 647–654.

[25] Price LE, Shea KD, Gephart SM (2015). The Veterans Affairs’s Corporate Data Warehouse: Uses and Implications for Nursing Research and Practice. Nurs Adm Q..

[26] Brenner LA, Breshears RE, Betthauser LM, Bellon KK, Holman E, Harwood JEF (2011). Implementation of a Suicide Nomenclature within Two VA Healthcare Settings. J Clin Psychol Med Settings..

[27] Sohn MW, Arnold N, Maynard C, Hynes DM. Accuracy and completeness of mortality data in the Department of Veterans Affairs. Popul Health Metrics. 2006;4(1):2. 10.1186/1478-7954-4-2. 10.1186/1478-7954-4-2 PMC1458356 16606453

[28] Furukawa TA, Cipriani A, Cowen PJ, Leucht S, Egger M, Salanti G (2019). Optimal dose of selective serotonin reuptake inhibitors, venlafaxine, and mirtazapine in major depression: a systematic review and dose-response meta-analysis. Lancet Psychiatry..

[29] Hayasaka Y, Purgato M, Magni LR, Ogawa Y, Takeshima N, Cipriani A (2015). Dose equivalents of antidepressants: Evidence-based recommendations from randomized controlled trials. J Affect Disord..

[30] WHO Collaborating Centre for Drug Statistics Methodology , Folkehelseinstituttet (Noruega). Guidelines for ATC classification and DDD assignment 2011. Oslo: WHO Collaborating Centre for Drug Statistics Methodology : Norwegian Institute of Public Health; 2010. OCLC: 804776084.

[31] WHO. WHO collaborative centre for drug statistics methodology ATC/DDD system. https://www.whocc.no/atc_ddd_index/. Accessed 19 Jan 2022.

[32] Coupland C, Hill T, Morriss R, Arthur A, Moore M, Hippisley-Cox J. Antidepressant use and risk of suicide and attempted suicide or self harm in people aged 20 to 64: cohort study using a primary care database. BMJ. 2015;350(feb18 32):h517. 10.1136/bmj.h517. 10.1136/bmj.h517 PMC4353276 25693810

[33] Coupland C, Hill T, Morriss R, Moore M, Arthur A, Hippisley-Cox J (2018). Antidepressant use and risk of adverse outcomes in people aged 20–64 years: cohort study using a primary care database. BMC Med..

[34] Jakubovski E, Varigonda AL, Freemantle N, Taylor MJ, Bloch MH (2016). Systematic Review and Meta-Analysis: Dose-Response Relationship of Selective Serotonin Reuptake Inhibitors in Major Depressive Disorder. Am J Psychiatr..

[35] Suehs B, Argo T, Bendele BSD, Crismon ML, Trivedi MH, Kurian B. Texas medication algorithm project procedural manual. Major depressive disorder algorithms Texas: Texas Department of State Health Services; 2008.

[36] McCarthy JF, Cooper SA, Dent KR, Eagan AE, Matarazzo BB, Hannemann CM (2021). Evaluation of the Recovery Engagement and Coordination for Health-Veterans Enhanced Treatment Suicide Risk Modeling Clinical Program in the Veterans Health Administration. JAMA Netw Open..

[37] Bayes AJ, Parker GB (2018). Comparison of guidelines for the treatment of unipolar depression: a focus on pharmacotherapy and neurostimulation. Acta Psychiatr Scand..

[38] American Psychiatric Association. Practice guideline for the treatment of patients with major depressive disorder. 3rd ed. Washington: American Psychiatric Association; 2010. OCLC: 708239605.

[39] American Psychological Association. Clinical practice guideline for the treatment of depression across three age cohorts. 2019. Retrieved from https://www.apa.org/depression-guideline.

[40] Song M, Günther CW, Van Der Aalst WMP. Trace Clustering in Process Mining. In: Ardagna D, Mecella M, Yang J, editors. Business Process Management Workshops. vol. 17. Berlin, Heidelberg: Springer Berlin Heidelberg; 2009. p. 109–120. http://link.springer.com/10.1007/978-3-642-00328-8_11.

[41] De Koninck P, Vanden Broucke S, De Weerdt J. act2vec, trace2vec, log2vec, and model2vec: Representation Learning for Business Processes. In: Weske M, Montali M, Weber I, Vom Brocke J, editors. Business Process Management. vol. 11080. Cham: Springer International Publishing; 2018. p. 305–321. http://link.springer.com/10.1007/978-3-319-98648-7_18.

[42] Seeliger A, Luettgen S, Nolle T, Mühlhäuser M. Learning of Process Representations Using Recurrent Neural Networks. In: La Rosa M, Sadiq S, Teniente E, editors. Advanced Information Systems Engineering. vol. 12751. Cham: Springer International Publishing; 2021. p. 109–124. https://link.springer.com/10.1007/978-3-030-79382-1_7.

[43] Hu L, Yang Y, Tang Z, He Y, Luo X (2023). FCAN-MOPSO: An Improved Fuzzy-Based Graph Clustering Algorithm for Complex Networks With Multiobjective Particle Swarm Optimization. IEEE Trans Fuzzy Syst..

[44] Lu X, Tabatabaei SA, Hoogendoorn M, Reijers HA. Trace Clustering on Very Large Event Data in Healthcare Using Frequent Sequence Patterns. In: Hildebrandt T, Van Dongen BF, Röglinger M, Mendling J, editors. Business Process Management. vol. 11675. Cham: Springer International Publishing; 2019. p. 198–215. http://link.springer.com/10.1007/978-3-030-26619-6_14.

[45] Prodel M, Augusto V, Jouaneton B, Lamarsalle L, Xie X (2018). Optimal Process Mining for Large and Complex Event Logs. IEEE Trans Autom Sci Eng..

[46] Fournier-Viger P, Faghihi U, Nkambou R, Nguifo EM. CMRules: Mining sequential rules common to several sequences. Knowl-Based Syst. 2012;25(1):63–76. 10.1016/j.knosys.2011.07.005.

[47] Fournier-Viger P, Wu CW, Tseng VS, Nkambou R. Mining Sequential Rules Common to Several Sequences with the Window Size Constraint. In: Hutchison D, Kanade T, Kittler J, Kleinberg JM, Mattern F, Mitchell JC, et al., editors. Advances in Artificial Intelligence. vol. 7310. Berlin, Heidelberg: Springer Berlin Heidelberg; 2012. p. 299–304. http://link.springer.com/10.1007/978-3-642-30353-1_27.

[48] Lo D, Khoo SC, Wong L. Non-redundant sequential rules—Theory and algorithm. Inf Syst. 2009;34(4–5):438–53. 10.1016/j.is.2009.01.002.

[49] Tan PN, Kumar V, Srivastava J (2004). Selecting the right objective measure for association analysis. Inf Syst..

[50] Gansner ER, Koutsofios E, North SC, Vo KP (1993). A technique for drawing directed graphs. IEEE Trans Softw Eng..

[51] Stübner S, Grohmann R, Greil W, Zhang X, Müller-Oerlinghausen B, Bleich S, et al. Suicidal Ideation and Suicidal Behavior as Rare Adverse Events of Antidepressant Medication: Current Report from the AMSP Multicenter Drug Safety Surveillance Project. Int J Neuropsychopharmacol. 2018;21(9):814–821. 10.1093/ijnp/pyy048. 10.1093/ijnp/pyy048 PMC6119288 29939264

[52] Luo Y, Kataoka Y, Ostinelli EG, Cipriani A, Furukawa TA. National prescription patterns of antidepressants in the treatment of adults with major depression in the US between 1996 and 2015: a population representative survey based analysis. Front Psychiatry. 2020;11. 10.3389/fpsyt.2020.00035. 10.3389/fpsyt.2020.00035 PMC7033625 32116850

